# Performance and Inter-Limb Asymmetry in Relation to Peak Height Velocity and Injury-Related Variables in Adolescent Male Soccer Players

**DOI:** 10.3390/sports14060227

**Published:** 2026-06-02

**Authors:** Alberto Roso-Moliner, Rafael Albalad-Aiguabella, Demetrio Lozano, Borja Sancho-Monllor, Oscar Villanueva-Guerrero, José Luis Arjol-Serrano

**Affiliations:** Health Sciences Faculty, Universidad San Jorge, Autovía A23 km 299, Villanueva de Gállego, 50830 Zaragoza, Spain; aroso@usj.es (A.R.-M.); ralbalad@usj.es (R.A.-A.); bsancho@usj.es (B.S.-M.); ovillanueva@usj.es (O.V.-G.); jlarjol@usj.es (J.L.A.-S.)

**Keywords:** change of direction, peak height velocity, physical performance, sport injuries, adolescent footballers

## Abstract

Adolescent footballers exhibit smaller change of direction (COD) deficits than adults, suggesting distinct biomechanical profiles; however, the role of physical performance variables in COD, considering maturation and injury-related factors, remains poorly understood. This study aimed to examine the relationships between sprint, jump, and COD performance, maturation status, and injury-related variables in young male footballers. Fifty-six national-level players (age: 16.67 ± 0.86 years) performed unilateral vertical and horizontal jump tests, 20 m linear sprint tests, and 180° COD assessments. Maturation status was estimated using peak height velocity (PHV), and injury incidence and severity were recorded over one competitive season. Associations were observed between PHV and sprint performance during the initial acceleration phase (0–10 m; *p* < 0.01). Unilateral jump measures were associated with sprint and COD performance, whereas inter-limb asymmetries showed limited associations with performance outcomes. Horizontal jump performance was also associated with the percentage-based COD deficit (%CODD). With respect to injury-related variables, injury incidence was associated with countermovement jump (CMJ) measures, with greater CMJ asymmetry being associated with higher injury incidence, while both unilateral CMJ variables were retained in the regression model. Overall, these findings suggest that biological maturation and unilateral neuromuscular performance may be relevant factors associated with youth football performance, whereas inter-limb asymmetry appears to play a more limited role; CMJ-related measures may warrant further consideration in relation to injury incidence in adolescent footballers.

## 1. Introduction

Repeated high-intensity actions such as accelerations, decelerations, linear sprints, and changes of direction (COD) are key factors in an intermittent sport such as soccer [1,2]. Among these, the ability to change direction quickly, efficiently, and with a reduced risk of injury allows players to respond to the tactical demands of the game, such as beating an opponent, regaining ball possession, or adapting to offensive and defensive transitions [3]. Performance in these COD actions depends on multiple neuromuscular and biomechanical factors, including linear speed, lower-limb strength and power, as well as the ability to apply force effectively across different planes of movement [4,5]. In recent years, several studies have examined the relationship between performance in sprint and/or jump tests and COD ability in soccer players, showing that acceleration during the first meters of a sprint and the ability to generate horizontal force are associated with the execution of rapid changes of direction [6,7].

Vertical and horizontal jump tests are also commonly used to assess lower-limb power and its relationship with performance in explosive game actions [8]. These tests, together with linear sprint and COD tests, constitute common assessment tools in soccer due to their strong reliability, sensitivity, and ease of application in field environments [9,10]. Furthermore, previous research has shown that variables such as sprinting, jumping, and changes of direction are responsive to neuromuscular and plyometric training interventions, highlighting their potential relevance in the development of physical performance in soccer players [11].

In youth populations, the evaluation of physical capacities must be interpreted considering the processes of growth and biological maturation. During adolescence, significant hormonal, neuromuscular, and anthropometric changes occur that may influence the development of physical qualities such as strength, power, and speed [12]. In this context, the moment of maximum growth rate, known as peak height velocity (PHV), has been associated with significant improvements in sprint performance and lower-limb power [13,14]. Furthermore, recent research has indicated that the sprint force–velocity profile undergoes modifications throughout development and during the competitive season in young soccer players, showing progressive increases in force production and maximum velocity in older age categories [15]. However, accelerated growth may generate alterations in motor coordination and in the mechanical efficiency of movement, affecting complex skills such as COD. In this regard, maturational status may be associated with both physical performance and training adaptations in adolescent soccer players [10].

On the other hand, the analysis of symmetry between lower limbs has gained increasing relevance in both sports’ performance and injury prevention. Asymmetries in unilateral actions such as jumps or changes of direction may influence force production capacity and may be associated with injury occurrence in sports characterized by repeated multidirectional movements [16]. Recent research has also found relationships between performance in unilateral jumps, acceleration, and the change of direction deficit (%CODD), suggesting that these variables may provide useful information for training planning and injury prevention in soccer players [7]. In addition, injury occurrence in soccer is commonly reported as the number of injuries per 1000 h (h) of exposure, which provides a standardized method for comparing injury rates across training and match contexts [17]. From a neuromuscular perspective, reduced levels of unilateral strength and power may compromise movement efficiency and limb stability during high-intensity actions such as sprinting, decelerating, and changing direction [18]. Likewise, although the available evidence is inconsistent, inter-limb asymmetries greater than 10% have frequently been considered a practically relevant threshold in sport settings, as they may reflect functional imbalances that may be associated with increased mechanical stress and injury susceptibility [19]. Nevertheless, recent reviews have highlighted that this threshold should be interpreted with caution, since its evidence base is not definitive and the relationship between asymmetry and injury risk remains inconsistent [20]. Therefore, analyzing injury incidence together with unilateral neuromuscular performance and inter-limb asymmetry may provide useful information for injury monitoring and preventive decision-making in adolescent soccer players.

Despite the growing interest in this area, there is still limited scientific evidence analyzing the relationship between performance in sprint and jump tests, maturational status, and injuries in adolescent soccer players simultaneously. More specifically, few studies have simultaneously examined sprint and jump performance, maturational status, inter-limb asymmetries, and injury incidence in young male footballers. Understanding these interactions may help to optimize training programs and reduce injury risk during critical stages of development. Therefore, the aim of this study was to examine the relationships between sprint, jump, and change of direction performance metrics, maturation status, and injury-related variables in young male footballers. It was hypothesized that sprint and jump performance metrics would be significantly associated with change of direction performance, that biological maturation would be associated with sprint performance outcomes, and that inter-limb asymmetries would have limited relevance in explaining both performance and injury-related outcomes.

## 2. Materials and Methods

### 2.1. Participants

Fifty-six well-trained adolescent football players (age: 16.67 ± 0.86 years; height: 177.17 ± 6.48 cm; body mass: 67.31 ± 7.10 kg) agreed to participate in the study. An a priori power analysis was conducted using G*Power (version 3.1.9.3, Düsseldorf, Germany) for a linear multiple regression model (fixed model, R^2^ deviation from zero). Based on an alpha level of 0.05, a desired statistical power of 0.80, and two predictors, the available sample size (*n* = 56) was sufficient to detect a moderate-to-large effect size (f^2^ ≈ 0.18–0.20). Players were eligible for inclusion if they were male adolescent footballers aged between 15 and 18 years, registered in a professional club academy, and had a minimum of four years of systematic football training experience. Additional inclusion criteria required regular participation in team training sessions and competitive matches throughout the season. Players were excluded if they presented any musculoskeletal injury or medical condition at the time of testing that could affect physical performance, had sustained an injury resulting in training or match absence during the testing period, or failed to complete all testing procedures. Goalkeepers were also excluded due to the specific physical demands of their playing position. All participants had been involved in club-level football training for at least four years, completing four weekly technical–tactical field-based training sessions of 90 min each and one competitive match per week. In addition, they performed one weekly 35 min strength training session focused on coordinative work and the development of muscular strength, with the aim of maintaining and improving physical fitness. Two teams from the same football club participated in the study. Both teams belonged to the youth academy of a professional football club and competed in national categories. Written informed consent was obtained from the parents or legal guardians of all participants. The study was conducted in accordance with the ethical principles of the Declaration of Helsinki (2013) and was approved by the local ethics committee (CP19/039, CEICA, Zaragoza, Spain).

### 2.2. Procedures

All physical fitness assessments were conducted on the same day and performed in a fixed sequence: unilateral horizontal and vertical jump tests, linear sprint testing, and CODs assessments. Participants were instructed to refrain from high-intensity physical activity for at least 48 h prior to testing to minimize residual fatigue. They were also advised to avoid caffeine and other stimulants (e.g., energy drinks or dietary supplements) that could influence performance, as well as maintain appropriate nutrition and optimal hydration during the 48 h preceding the assessments. All players were familiar with the testing procedures, having previously completed each test on at least five occasions. To ensure adequate recovery and consistency across tests, a standardized rest period of 1 min (jumps) or 3 min (sprints and CODs) was provided between each assessment. Athletic footwear was used for the jumping tests, whereas soccer boots were worn for the linear sprint and COD tests. Before testing, participants completed a standardized warm-up based on the raise, activate, mobilize, and potentiate (RAMP) framework [21]. The activation phase included dynamic exercises targeting the primary muscle groups involved in the tests, followed by a mobilization phase consisting of dynamic stretching aimed at enhancing joint mobility and flexibility. These included the hip extensors and flexors (gluteus maximus, iliopsoas, rectus femoris), knee extensors and flexors (quadriceps femoris, hamstrings), ankle plantar flexors (gastrocnemius and soleus), and hip stabilizers (adductors and abductors). The warm-up concluded with a potentiation phase designed to prepare the neuromuscular system through specific drills that replicated the movement patterns and intensities required during testing.

#### 2.2.1. Unilateral Countermovement Jump Test

The test was performed twice, with a 1 min rest interval between trials, and the best performance was retained for analysis. Participants executed a unilateral countermovement jump with their hands placed on the hips, while the contralateral leg was flexed at 90° at both the hip and knee for the left and right legs (CMJL and CMJR, respectively). Jump performance was assessed using the Optojump system, version 1.13.24.0 (Microgate, Bolzano, Italy). A trial was deemed invalid if proper technique was not maintained, such as failure to keep the hands on the hips or not landing on the same leg used for take-off. Movement of the non-supporting leg during the jump was allowed; however, upon landing, participants were required to maintain balance on the jumping leg for 3 s to confirm a valid attempt [22]. The test demonstrated excellent reliability, with an intraclass correlation coefficient (ICC) of 0.92 (>0.90) [23] and a coefficient of variation (CV) of 3.5%.

#### 2.2.2. Unilateral Horizontal Jump Test

Performance in the left and right unilateral horizontal jumps (HJL and HJR) was assessed by measuring jump distance with a standard tape measure with up to 0.01 m accuracy. Each participant performed two trials per leg, with a 1 min rest interval between attempts, and the best performance was retained for analysis. Use of the free leg to generate momentum was permitted, and participants were required to take off and land on the same leg. Only trials in which participants were able to maintain balance and hold the final landing position for 3 s were considered valid [7]. The test demonstrated excellent reliability, with an ICC of 0.90 (>0.90) [23] and a CV of 2.5%.

#### 2.2.3. The 20 m Sprint Test

Running speed was evaluated using a 20 m linear sprint, with split times recorded over 0–10 m and 10–20 m [24]. Sprint times were measured using dual-beam photocells (Witty, Microgate, Bolzano, Italy). Participants adopted a two-point staggered stance, with the front foot positioned 0.5 m behind the first timing gate. The timing gates were placed at a height of 0.75 m and separated laterally by 1.5 m [25]. Each participant completed two sprint trials, separated by at least 3 min of passive recovery, and the fastest trial was selected for analysis [22]. The test demonstrated excellent reliability, with an ICC of 0.93 (>0.90) [23] and a CV of 1.5%.

#### 2.2.4. The 180° COD Test

A 10 m shuttle sprint test was performed to assess COD ability. Participants sprinted from the start/finish line to the 5 m mark, touched the line with either foot, executed a 180° turn, and sprinted back to the finish line. A trial was considered valid only if the outside foot crossed the finish line. The starting position required the leading foot to be placed 0.5 m behind the first timing gate. Sprint times were recorded using dual-beam photocell timing gates positioned at a height of 0.75 m and separated by 1.5 m (Witty, Microgate, Bolzano, Italy). Each participant performed two trials, one initiating with the left foot and the other with the right, in an alternating order, with 3 min of passive recovery between attempts. The fastest time for each starting foot was retained for analysis. The analyzed variables included the 180° change-of-direction performance with the left (COD180L) and right (COD180R) legs. Test reliability was good, with an ICC of 0.82 (0.75–0.90) [23] and a CV of 2%. The percentage-based *COD* deficit was calculated using the following formula [7,22]:

%*CODD* = ((*COD time* − 10 m *sprint time*)/10 m *sprint time*) × 100

#### 2.2.5. Assessment of Maturity

Relative age at peak height velocity (PHV), which identifies the period of maximum growth rate in stature during adolescence, is one of the most commonly used indicators of biological maturity in longitudinal studies at this stage of development, as it provides a precise reference point for the moment of peak growth during maturation [26]. According to the proposal by Mirwald et al. [27] this simple and non-invasive method allows the estimation of maturity offset (i.e., age relative to PHV) using a predictive equation that includes anthropometric variables together with chronological age and is considered a valid approach for classifying individuals’ maturity status.

#### 2.2.6. Injury Incidence

The total number of injuries sustained by both teams during the 2024–2025 competitive season was recorded, considering all exposure events (training sessions and matches), in order to calculate injury incidence. The injury rate was estimated as the number of injuries recorded per 1000 h of exposure [28].

#### 2.2.7. Inter-Limb Asymmetry Calculation

Inter-limb asymmetry was calculated using the following equation:

*Asymmetry* (%) = (|*Higher-performing limb* − *Lower-performing limb*|/*Higher-performing limb*) × 100

This method was selected due to its widespread use in applied sport science research and its capacity to provide magnitude-based interpretation independent of limb dominance [29].

### 2.3. Statistical Analyses

Data are presented as mean ± SD. Within-session reliability was assessed by coefficient of variation (CV) and intraclass correlation (ICC) using a spreadsheet specially designed for calculations. The Shapiro–Wilk test was employed to evaluate normality, indicating that all variables followed a normal distribution, except for inter-limb asymmetries. Multiple linear regression models were applied, using a backward stepwise elimination approach, with PHV times and injuries as dependent variables. The independent variables were unilateral vertical and horizontal jumps, 0–10 m, 10–20 m and 0–20 m sprints, and CODs. In the backward procedure, variables with *p*-values > 0.05 were removed from the model. Relationships between PHV and injuries with the other variables were evaluated through Pearson’s product moment coefficient and lineal regression. According to Schober, Boer, and Schwarte (2018) [30], the strength of a correlation coefficient (r) can be interpreted using a general guideline based on its absolute value: coefficients between 0.00 and 0.10 indicate negligible correlation, 0.10 to 0.39 weak correlation, 0.40 to 0.69 moderate correlation, 0.70 to 0.89 strong correlation, and 0.90 to 1.00 very strong correlation. Statistical evaluations were conducted with the aid of SPSS (version 28, IBM, New York, NY, USA) and Microsoft Excel (version 2016, Microsoft Corp., Redmond, WA, USA).

Given the limited sample size, regression models were developed using a theory-driven and parsimonious approach. Only variables showing significant bivariate correlations and clear biomechanical relevance were entered into regression analyses. Multicollinearity was assessed using the Variance Inflation Factor (VIF), and predictors with VIF values > 5 were removed. Redundant sprint variables were not included within the same model. For all regression coefficients, 95% confidence intervals (CI) are reported. These methodological decisions were adopted to minimize overfitting and enhance model stability.

## 3. Results

Table 1 summarizes the descriptive characteristics of the participants. Anthropometric measures, indices of biological maturation, and injury incidence are reported as means and standard deviations in order to provide a comprehensive overview of the sample’s physical profile and maturational status.

Injury descriptive data are presented in Table 2. A total of 43 injuries were recorded during the study period, of which 12 were classified as minor (≤7 days), 23 as moderate (8–28 days), and 8 as severe (>28 days). The overall injury incidence was 7 injuries per 1000 h of exposure.

Descriptive statistics for physical performance and inter-limb asymmetries are shown in Table 3. Unilateral countermovement jump height was similar between the right (21.27 ± 3.06 cm) and left limbs (21.41 ± 3.12 cm), with a mean asymmetry of 6.61 ± 4.28%, while unilateral horizontal jump performance also showed comparable values between limbs (right: 175.96 ± 10.70 cm; left: 176.39 ± 10.10 cm) and lower asymmetry (3.02 ± 2.30%). Sprint performance over 20 m resulted in mean times of 1.71 ± 0.07 s (0–10 m), 1.27 ± 0.06 s (10–20 m), and 2.97 ± 0.11 s (0–20 m). Change of direction times were slightly faster to the left (2.45 ± 0.13 s) than to the right (2.49 ± 0.13 s), with an inter-limb asymmetry of 3.69 ± 2.84%, whereas the percentage-based change of direction deficit showed greater variability between directions (right: 45.11 ± 7.56%; left: 47.78 ± 8.52%) and the highest asymmetry values (11.40 ± 7.40%).

Pearson correlation coefficients between physical performance variables, maturation indicators, and injury incidence are presented in Table 4. PHV was significantly and negatively correlated with maturation differences (r = −0.435, *p* < 0.01) and sprint performance over 10–20 m (r = −0.425, *p* < 0.01). Significant associations were observed between unilateral jump performances, with strong correlations between CMJL and CMJR (r = 0.832, *p* < 0.001) and between HJL and HJR (r = 0.781, *p* < 0.001). Unilateral countermovement jump performance showed moderate negative correlations with sprint intervals (CMJL with 0–10 m: r = −0.359, *p* < 0.05; CMJL with 10–20 m: r = −0.455, *p* < 0.01; CMJR with 10–20 m: r = −0.328, *p* < 0.05). Strong positive correlations were found among sprint segments (0–10 m with 10–20 m: r = 0.615, *p* < 0.001; 0–10 m with 0–20 m: r = 0.917, *p* < 0.001; 10–20 m with 0–20 m: r = 0.878, *p* < 0.001). COD times to the left and right were also significantly correlated (r = 0.521, *p* < 0.01), and a very strong association was observed between %CODDR and %CODDL (r = 0.943, *p* < 0.001). No significant correlations were identified between performance variables and injury incidence (*p* > 0.05).

Pearson correlation coefficients between asymmetry indices, maturation variables, and injury incidence are presented in Table 5. No significant relationships were observed between PHV or maturation differences and any asymmetry measures (*p* > 0.05). Injury incidence showed a significant positive correlation with countermovement jump asymmetry (r = 0.468, *p* < 0.05), while no significant associations were found between injury incidence and horizontal jump or change of direction asymmetries (*p* > 0.05).

Linear regression analyses examining the relationships between biological maturation, injury incidence, and physical performance variables are presented in Table 6. The models including PHV (R = 0.732; R^2^ = 0.535; *p* = 0.483) and maturation differences (R = 0.589; R^2^ = 0.347; *p* = 0.867) were not statistically significant, and no individual performance variables showed significant associations within these models (*p* > 0.05). The model including injury incidence demonstrated a higher explained variance (R = 0.863; R^2^ = 0.744), although the overall model did not reach statistical significance (*p* = 0.082). Within this model, significant associations were observed for unilateral countermovement jump performance, with CMJL positively associated (β = 0.927, *p* = 0.017) and CMJR negatively associated (β = −0.908, *p* = 0.049) with injury incidence, while no significant relationships were identified for sprint, horizontal jump, change of direction, or asymmetry variables (*p* > 0.05).

Linear regression analyses examining the relationships between maturation variables, injury incidence, and asymmetry indices are presented in Table 7. The regression models including PHV (R = 0.419; R^2^ = 0.175; *p* = 0.458) and maturation differences (R = 0.472; R^2^ = 0.222; *p* = 0.335) were not statistically significant, and none of the asymmetry measures (% CMJ, % HJ, or % COD) showed significant associations within these models (*p* > 0.05).

## 4. Discussion

The present study was conducted to address the limited evidence simultaneously examining biological maturation, unilateral neuromuscular performance, inter-limb asymmetry, change of direction performance, and injury-related variables in adolescent male soccer players. Given the multifactorial nature of physical development and injury occurrence during adolescence, understanding these associations may help practitioners interpret performance testing outcomes within a maturational and injury-monitoring context. Therefore, this study aimed to examine the relationships between sprint, jump, and change of direction performance metrics, maturation status, and injury-related variables in young male footballers. Overall, the initial expectations were partially supported. The main findings of the present study suggest that biological maturation, estimated through variables related to peak height velocity (PHV), was moderately associated with sprint performance over the 10–20 m split, rather than with all sprint phases. Unilateral neuromuscular performance, particularly in vertical and horizontal jump tests showed weak-to-moderate associations with linear sprint performance. In addition, only selected associations were observed between sprint performance, COD variables, and percentage-based COD deficit measures, and these should be interpreted according to their magnitude. Regarding injury-related outcomes, a significant association was found between injury incidence and CMJ asymmetry, with greater inter-limb asymmetry in the countermovement jump being related to greater injury incidence. Furthermore, the asymmetry-based regression model explained 46.4% of the variance in injury incidence, indicating that although the model was statistically significant, more than half of the variance remained unexplained. In contrast, the performance-based regression model including injury incidence as the dependent variable did not reach statistical significance, despite significant individual coefficients for CMJL and CMJR; therefore, these findings should be interpreted cautiously and considered exploratory. Overall, the results indicate that biological maturation and unilateral neuromuscular capacity may provide complementary information for interpreting functional performance, while inter-limb asymmetry showed limited associations with performance outcomes. However, given the correlational nature of the study, these findings should be interpreted as associations rather than causal or predictive relationships.

First, the association observed between PHV-related variables and sprint performance over 0–10 m (r = −0.425; *p* = 0.01; moderate correlation) indicates a statistically significant moderate association between maturational status and initial acceleration during developmental stages, although this association should not be interpreted causally. This finding is consistent with previous literature, as Fernández-Galván et al. [31] reported differences between maturity groups in young soccer players, with faster sprint times and more favorable mechanical profiles as biological maturation progressed. Similarly, McCunn et al. [32] showed that the relationship between maturity offset and sprint performance over 15 m could reach moderate magnitude in age groups such as U15 (r = −0.62). In this context, the moderate magnitude of the association observed in the present study suggests that maturation may be related to, but does not fully explain, short-distance sprint performance.

A noteworthy finding was the negative association between PHV and 0–10 m sprint performance, suggesting that later maturing players tended to exhibit faster acceleration over short distances. This result contrasts with the substantial body of evidence indicating that earlier maturation is generally associated with greater speed and power performance in youth soccer, largely due to advanced neuromuscular and morphological development [26,33,34]. However, this association should be interpreted cautiously. Short-distance sprint performance in adolescent players is influenced not only by biological maturation but also by technical execution, motor coordination, and task familiarity, which may attenuate expected maturation-related advantages. In addition, cohort-specific selection effects cannot be excluded, as later maturing players who remain in elite pathways may represent a subgroup with greater movement efficiency or compensatory qualities. Therefore, this finding does not necessarily contradict existing maturation theory but rather highlights the multifactorial nature of acceleration performance and the need to consider biological, technical, and contextual factors when interpreting maturation–performance relationships in youth athletes.

From a physiological perspective, this pattern may be partly related to the neuromuscular and anthropometric changes associated with growth and maturation, as prior studies have demonstrated significant associations between PHV, skeletal age, lean mass, and muscle strength and power variables in adolescent athletes [35]. Therefore, the present findings support the need to interpret short sprint performance in youth athletes not only based on chronological age but also considering biological age [36]. On the other hand, the associations observed between unilateral CMJ performance and sprint outcomes suggest a potential shared functional basis between rapid force production capacity and linear speed performance [6,37]. In the present study, CMJL was significantly associated with all sprint phases analyzed (r = −0.359 to −0.455; *p* = 0.008 to 0.04; weak-to-moderate correlations), whereas CMJR was only related to total sprint time over 0–20 m (r = −0.349; *p* = 0.047; weak correlation). These findings suggest that absolute unilateral performance may provide complementary information when interpreting sprint performance in adolescent soccer players [38]. In line with this, Michailidis [39] reported in U16 male soccer players that asymmetries identified through single-leg CMJ and COD tests were not associated with acceleration or linear sprint performance, indicating that the percentage difference between limbs does not necessarily explain performance, particularly when asymmetry magnitudes are relatively small. Therefore, the present results suggest that unilateral neuromuscular capacity may be more closely associated with sprint performance than asymmetry magnitude alone in young athletes.

Another relevant finding was the significant relationship between CMJR and HJR (r = 0.340; *p* = 0.028; weak correlation). However, the correlations between horizontal jump performance and %CODDR were not statistically significant in the present study (HJL: r = 0.049, *p* = 0.825; HJR: r = 0.156, *p* = 0.479), and therefore no firm conclusion should be drawn regarding this specific relationship. This pattern suggests that the ability to generate explosive force during unilateral actions may share common neuromuscular characteristics across both vertical and horizontal force vectors, although the magnitude of these associations was generally small. From an applied perspective, this may be relevant, as horizontal force production may be related to tasks involving acceleration, deceleration, and repositioning of the center of mass, all of which are essential for effective change of direction performance. In this regard, Lin et al. [40] reported that horizontal jump tests show moderate to very large associations with sprint performance, both during the acceleration phase and at maximal velocity. Similarly, Lockie et al. [41] demonstrated that players with superior vertical jump performance tend to achieve faster COD times. Moreover, a recent meta-analysis reported that plyometric training induces moderate improvements in jump and COD performance, and smaller improvements in sprint performance, further supporting the notion that these physical capacities are functionally interconnected [42]. Similarly, the significant correlations observed between the different sprint phases (r = 0.615 to 0.917; *p* < 0.001; moderate-to-very strong correlations) and between 0–20 m sprint performance and %CODDR (r = 0.391; *p* = 0.048; weak correlation) suggest that linear speed and COD efficiency are partly related, rather than fully independent capacities. Previous research has shown that acceleration during the first 10 m of a sprint is associated with performance in 180° COD tests, accounting for between 28% and 50% of the variance in performance (*p* < 0.01) [7]. Although COD performance involves specific components such as deceleration, postural control, and reacceleration, it shares key mechanical determinants with linear sprinting, particularly the ability to produce horizontal force rapidly and effectively [43]. In this context, Loturco et al. [44] reported that soccer players with greater maximal acceleration not only sprint faster and jump higher, but also reach greater velocities during COD actions, although they may have exhibited a greater COD deficit, likely due to the greater inertia associated with superior approach velocities. Furthermore, the associations observed between COD180L and COD180R (r = 0.521; *p* = 0.001; moderate correlation) and between %CODDL and %CODDR (r = 0.943; *p* < 0.001; very strong correlation) were expected from a conceptual standpoint, as the CODD represents the additional time cost required to execute a directional change relative to linear sprinting [45].

Regarding injury-related outcomes, injury incidence showed a significant positive correlation with CMJ asymmetry (r = 0.468, *p* = 0.018; moderate correlation), suggesting that players with greater inter-limb differences in countermovement jump performance tended to present a greater number of injuries within this sample. Moreover, the regression model including asymmetry variables and injury incidence was statistically significant (R = 0.681; R^2^ = 0.464; ANOVA *p* = 0.012), with CMJ asymmetry showing a significant association within the model (*p* = 0.002). However, because this model explained less than 46.4% of the variance, the practical strength of this association should not be overstated. The performance-based regression model for injury incidence did not reach statistical significance (R = 0.863; R^2^ = 0.744; ANOVA *p* = 0.082), although CMJL and CMJR showed significant individual coefficients. Therefore, these regression findings should be interpreted as exploratory and hypothesis-generating rather than confirmatory. A recent systematic review has highlighted that prospective evidence on the relationship between inter-limb asymmetries and sports injury remains highly inconsistent, which limits the ability to draw firm conclusions about their predictive value [20]. In line with this, Raya-González et al. [46] reported that strength, jump, and COD asymmetries were not significantly associated with physical performance in elite academy soccer players. Similarly, Michailidis [39] found no consistent relationships between jump and COD asymmetries and key performance variables. This interpretation is further supported by recent studies indicating that asymmetries alone do not consistently predict poorer performance or increased injury risk [19]. However, the available literature is not uniform, as some studies have reported that greater functional asymmetries are associated with greater injury incidence. For example, Fort-Vanmeerhaeghe et al. [22] showed that greater vertical jump asymmetries, together with reduced physical performance, were associated with increased injury incidence in youth team-sport athletes. Taken together, these findings suggest that CMJ asymmetry, and to a lesser extent unilateral CMJ performance, may provide complementary information when interpreting injury incidence in this sample. Nevertheless, these results should be interpreted cautiously, as the present analyses identify statistical associations and do not establish causality or confirm the predictive value of CMJ asymmetry for future injury risk.

From a practical perspective, the findings of the present study may support the inclusion of maturational status, short sprint performance, and unilateral vertical and horizontal jump assessments in the regular monitoring of young athletes. PHV appears to be particularly useful for contextualizing performance during the initial acceleration phase, while unilateral assessments may offer more precise information regarding the neuromuscular function of each limb and its potential relationship with sprint, COD performance, and injury-related outcomes. In particular, the regular monitoring of unilateral CMJ performance and inter-limb asymmetries may help to identify neuromuscular characteristics associated with injury incidence. Therefore, the combined evaluation of absolute unilateral jump performance and asymmetry magnitude may assist practitioners in identifying athletes who may present greater injury incidence within similar contexts and in designing more individualized injury prevention strategies. At the same time, the limited explanatory role of inter-limb asymmetries in functional performance outcomes suggests that small between-limb differences should not be interpreted in isolation or overemphasized in the absence of meaningful performance impairments or a relevant injury history. Consequently, applied assessment should move beyond the isolated interpretation of asymmetry percentages and instead adopt a more comprehensive approach that considers absolute functional performance and its progression throughout growth and maturation.

An important limitation of this study is the relatively small sample size for multivariate statistical modeling. Although regression analyses were conducted using parsimonious models, the study was not sufficiently powered for complex multivariable prediction. Consequently, regression findings should be interpreted as exploratory, and future studies with larger samples are required to confirm these relationships. Additional limitations are also acknowledged. First, the cross-sectional design and the assessment of players at a single time point during the season do not allow changes in physical performance, asymmetry, or injury-related indicators to be examined across the competitive year. These variables may vary over time due to growth, training, fatigue, and match demands, and therefore the findings should be interpreted as reflecting one specific stage of the season rather than the players’ seasonal profile. Second, all participants were recruited from a single club with its own training methodology, which may limit the applicability of the findings to athletes from other settings, particularly those with different training backgrounds or reduced performance levels. Third, the study focused on performance and asymmetry measures, but other factors related to injury risk, such as training load, fatigue, recovery status, and match exposure, were not included. Monitoring these variables may help to better understand injury risk and improve the interpretation of physical performance and asymmetry data. Future research should use longitudinal designs, include repeated assessments across the season, and consider a wider range of contextual variables in order to provide a clearer understanding of the relationships between maturation, neuromuscular performance, asymmetry, and risk in youth athletes.

## 5. Conclusions

The present study indicates that the strongest associations were mainly observed between closely related performance measures, particularly between sprint split times, bilateral jump performances, and percentage-based COD deficit variables. These findings suggest that sprint, jump, and COD derived measures provide complementary information for characterizing neuromuscular performance in adolescent male footballers. In contrast, inter-limb asymmetries showed limited associations with performance outcomes, indicating that asymmetry magnitude should not be interpreted in isolation when assessing functional capacity. Regarding injury-related outcomes, CMJ asymmetry showed a moderate association with injury incidence, suggesting that CMJ derived measures may offer useful complementary information within broader athlete monitoring strategies. However, given the correlational design of the study and the exploratory nature of the regression analyses, these findings should be interpreted as associations rather than evidence of causality or definitive injury prediction. From an applied perspective, practitioners should interpret maturation status, unilateral performance, and asymmetry measures together with contextual factors such as injury history, training load, fatigue, and match exposure.

## Figures and Tables

**Table 1:** Mean and standard deviation of descriptive data.

Variable	Mean ± SD
Age (y)	16.67 ± 0.86
Body Mass (kg)	67.31 ± 7.10
BMI	21.39 ± 1.30
PHV	14.36 ± 0.68
Maturation Differences	2.98 ± 0.85

Note: y: years; kg: kilograms; BMI: body mass index; PHV: peak height velocity; SD: standard deviation.

**Table 2:** Injuries descriptive data.

Variable	n
Total injuries	43
Minor (≤7 days)	12
Moderate (8–28 days)	23
Severe (>28 days)	8
Incidence of injuries (1000 h/exposure)	7

**Table 3:** Descriptive data for all physical performance and asymmetry tests.

Variable	Mean ± SD	Asymmetries (%)
CMJR (cm)	21.27 ± 3.06	6.61 ± 4.28
CMJL (cm)	21.41 ± 3.12	
HJR (cm)	175.96 ± 10.70	3.02 ± 2.30
HJL (cm)	176.39 ± 10.10	
20 m (0–10 m)	1.71 ± 0.07	
20 m (10–20 m)	1.27 ± 0.06	
20 m (0–20 m)	2.97 ± 0.11	
COD180R (s)	2.49 ± 0.13	3.69 ± 2.84
COD180L (s)	2.45 ± 0.13	
%CODDR (%)	45.11 ± 7.56	11.40 ± 7.40
%CODDL (%)	47.78 ± 8.52	

Note: SD: standard deviation; CMJR and CMJL: unilateral countermovement jumps with right and left legs; HJR and HJL: unilateral horizontal jump with right and left legs; 20 m: linear sprint of 20 m; COD180R and COD180L: 10 m shuttle sprint with one change of direction to right or left; %CODDR and %CODDL: percentage-based change of direction deficit to right or left and Sprint of 0–10 m.

**Table 4:** Pearson correlation coefficients (r) between all performance scores for each test.

	Maturation Differences	CMJL	CMJR	HJL	HJR	20 m (0–10 m)	20 m (10–20 m)	20 m (0–20 m)	COD180L	COD180R	%CODDR	%CODDL	Incidence Injuries (1000 h/exp)
PHV	−0.435	0.08	0.097	0.066	0.251	−0.425	−0.114	−0.317	−0.012	−0.121	0.015	−0.020	0.012
	0.003 **	0.63	0.55	0.682	0.114	0.01 **	0.508	0.06	0.943	0.474	0.946	0.927	0.955
Maturation Differences		0.122	0.128	0.36	0.214	0.124	−0.026	0.089	0.303	0.199	−0.030	0.050	0.112
		0.45	0.430	0.021	0.18	0.472	0.881	0.607	0.068	0.238	0.888	0.818	0.610
CMJL			0.832	0.268	0.226	−0.359	−0.455	−0.449	−0.155	0.018	−0.159	−0.187	0.120
			<0.001 **	0.086	0.15	0.04 *	0.008 **	0.009 **	0.374	0.917	0.481	0.404	0.586
CMJR				0.285	0.340	−0.3	−0.328	−0.349	−0.122	0.004	−0.089	−0.123	0.119
				0.068	0.028 *	0.89	0.062	0.047 *	0.485	0.982	0.694	0.587	0.590
HJL					0.781	−0.126	−0.243	−0.199	−0.043	−0.264	0.049	0.120	0.216
					<0.001 **	0.478	0.167	0.26	0.806	0.12	0.825	0.586	0.311
HJR						−0.256	−0.297	−0.304	−0.095	−0.325	0.156	0.153	0.035
						0.145	0.088	0.081	0.581	0.053	0.479	0.485	0.870
20 m (0–10 m)							0.615	0.917	0.0256	0.251	0.390 *	0.354	−0.033
							<0.001 **	<0.001 **	0.116	0.123	0.049	0.076	0.892
20 m (10–20 m)								0.878	0.306	0.218	0.267	0.255	−0.029
								<0.001 **	0.058	0.183	0.187	0.210	0.905
20 m (0–20 m)									0.31	0.262	0.391 *	0.361	−0.033
									0.055	0.106	0.048	0.070	0.893
COD180L										0.521	0.018	−0.011	−0.059
										0.001 **	0.929	0.955	0.799
COD180R											0.007	0.031	−0.183
											0.974	0.880	0.428
%CODDR												0.943 **	0.262
												0.000	0.346
%CODDL													0.293
													0.289

Note: PHV: peak height velocity; CMJR and CMJL: unilateral countermovement jumps with right and left legs; HJR and HJL: unilateral horizontal jump with right and left legs; 20 m: linear sprint of 20 m; COD180R and COD180L: 10 m shuttle sprint with one change of direction to right or left; %CODDR and %CODDL: percentage-based change of direction deficit to right or left and sprint of 0–10 m. ** *p* < 0.01. * *p* < 0.05.

**Table 5:** Pearson correlation coefficients (r) between all asymmetry, maturation and injuries.

	% Asymmetry CMJ	% Asymmetry HJ	% Asymmetry COD180
PHV	0.190	0.244	0.147
	0.259	0.151	0.537
Maturation Differences	−0.042	−0.234	0.338
	0.806	0.169	0.144
Incidence injuries (1000 h/exp)	0.468 *	0.330	0.263
	0.018	0.107	0.434

Note: PHV: peak height velocity; CMJ: unilateral countermovement jumps with right and left legs; HJ: unilateral horizontal jump; COD180: 10 m shuttle sprint with one change of direction to right or left; * *p* < 0.05.

**Table 6:** Model linear regression analysis between biological maturation, injury incidence, and physical performance measures.

Variables	R	R^2^	*p* ANOVA	Model	SC β	95% CI		*p*
						Inf	Sup	
PHV	0.732	0.535	0.483	CMJL	−0.218	−0.342	0.216	0.622
				CMR	0.098	−0.318	0.373	0.859
				HJL	0.196	−0.077	0.107	0.721
				HJR	0.211	−0.086	0.114	0.757
				20 m (0−10 m)	−0.652	−16.672	0.451	0.061
				20 m (10−20 m)	0.020	−12.492	13.173	0.953
				COD180L	−0.063	−5.698	4.883	0.866
				COD180R	0.418	−5.989	11.503	0.494
Maturation Differences	0.589	0.347	0.867	CMJL	0.228	−0.264	0.396	0.663
				CMR	−0.296	−0.492	0.325	0.654
				HJL	0.108	−0.100	0.117	0.868
				HJR	0.088	−0.112	0.124	0.913
				20 m (0−10 m)	0.402	−5.141	15.111	0.294
				20 m (10−20 m)	−0.273	−19.757	10.597	0.512
				COD180L	0.251	−4.628	7.887	0.570
				COD180R	−0.136	−11.238	9.451	0.849
Incidence injuries (1000 h/exp)	0.863	0.744	0.082	CMJL	0.927	0.122	0.953	0.017 *
				CMR	−0.908	−1.031	−0.002	0.049 *
				HJL	−0.062	−0.146	0.127	0.879
				HJR	0.530	−0.077	0.219	0.308
				20 m (0−10 m)	0.418	−2.307	23.198	0.097
				20 m (10−20 m)	−0.190	−25.536	12.692	0.467
				COD180L	−0.297	−11.764	3.997	0.294
				COD180R	0.192	−10.486	15.568	0.669

Note: PHV: peak height velocity; CMJR and CMJL: unilateral countermovement jumps with right and left legs; HJR and HJL: unilateral horizontal jump with right and left legs; 20 m: linear sprint of 20 m; COD180R and COD180L: 10 m shuttle sprint with one change of direction to right or left; SC β: standardized coefficient β; CI: confidence intervals; * *p* < 0.05; multicollinearity was assessed using the Variance Inflation Factor (VIF); only predictors with acceptable VIF values were retained in the final models (VIF < 5).

**Table 7:** Model linear regression analysis with measures of PHV, Incidence injuries (1000 h/exp) injuries.

Variables	R	R^2^	*p* ANOVA	Model	SC β	95% CI		*p*
						Inf	Sup	
PHV	0.419	0.175	0.458	% Asy CMJ	0.225	−0.059	0.141	0.394
				% Asy HJ	0.36	−0.088	0.425	0.18
				% Asy COD	0.096	−0.162	0.231	0.709
Maturation Differences	0.472	0.222	0.335	% Asy CMJ	0.092	−0.087	0.123	0.715
				% Asy HJ	−0.194	−0.368	0.171	0.445
				% Asy COD	0.411	−0.047	0.366	0.118
Incidence injuries (1000 h/exp)	0.681	0.464	0.012 *	% Asy CMJ	0.68	−0.241	0.197	0.002 *
				% Asy HJ	−0.038	0.374	1.320	0.834
				% Asy COD	0.168	−0.188	0.485	0.364

Note: PHV: peak height velocity; CMJ countermovement jumps; HJ: horizontal jump; SC β: standardized coefficient β; CI: confidence intervals; * *p* < 0.05; multicollinearity was assessed using the Variance Inflation Factor (VIF); only predictors with acceptable VIF values were retained in the final models (VIF < 5).

## Data Availability

The original contributions presented in this study are included in the article. Further inquiries can be directed to the corresponding author.
